# The Design of Anionic Surfactant-Based Amino-Functionalized Mesoporous Silica Nanoparticles and their Application in Transdermal Drug Delivery

**DOI:** 10.3390/pharmaceutics12111035

**Published:** 2020-10-29

**Authors:** Aliyah Almomen, Ahmed M. El-Toni, Mohammed Badran, Adel Alhowyan, Mohd Abul Kalam, Aws Alshamsan, Musaed Alkholief

**Affiliations:** 1 Department of Pharmaceutical Chemistry, College of Pharmacy, King Saud University, Riyadh 11495, Saudi Arabia; alalmomen@ksu.edu.sa; 2 Nanobiotechnology Unit, College of Pharmacy, King Saud University, Riyadh 11495, Saudi Arabia; makalam@KSU.EDU.SA; 3 King Abdullah Institute for Nanotechnology, King Saud University, Riyadh 11451, Saudi Arabia; aamohammad@KSU.EDU.SA; 4 Nanomaterials and Nanotechnology Department, Central Metallurgical Research and Development Institute (CMRDI), Helwan, Cairo 11865, Egypt; 5 Department of Pharmaceutics, College of Pharmacy, King Saud University, Riyadh 11495, Saudi Arabia; mbadran@KSU.EDU.SA (M.B.); adel-ali@KSU.EDU.SA (A.A.); 6 Department of Pharmaceutics, Faculty of Pharmacy, Al-Azhar University, Cairo 11865, Egypt

**Keywords:** mesoporous silica nanoparticles, amino functionalized, transdermal, 5-flurouracil, dexamethasone, melanoma

## Abstract

Melanoma remains the most lethal form of skin cancer and most challenging to treat despite advances in the oncology field. Our work describes the utilization of nanotechnology to target melanoma locally in an attempt to provide an advanced and efficient quality of therapy. Amino-functionalized mesoporous silica nanoparticles (MSN-NH_2_) were developed in situ through the utilization of anionic surfactant and different volumes of 3-aminopropyltriethoxysilane (APTES) as a co-structure directing agent (CSDA). Prepared particles were characterized for their morphology, particles size, 5-flurouracol (5-FU) and dexamethasone (DEX) loading capacity and release, skin penetration, and cytotoxicity in vitro in HT-144 melanoma cells. Results of transmission electron microscopy (TEM) and nitrogen adsorption–desorption isotherm showed that using different volumes of APTES during the functionalization process had an impact on the internal and external morphology of the particles, as well as particle size. However, changing the volume of APTES did not affect the diameter of formed mesochannels, which was about 4 nm. MSN-NH_2_ showed a relatively high loading capacity of 5-FU (12.6 ± 5.5) and DEX (44.72 ± 4.21) when using drug: MSN-NH_2_ ratios of 5:1 for both drugs. The release profile showed that around 83% of 5-FU and 21% of DEX were released over 48 h in pH 7.4. The skin permeability study revealed that enhancement ratio of 5-Fu and DEX using MSN-NH_2_ were 4.67 and 5.68, respectively, relative to their free drugs counterparts. In addition, the accumulation of drugs in skin layers where melanoma cells usually reside were enhanced approximately 10 times with 5-FU and 5 times with DEX when delivering drugs using MSN-NH_2_ compared to control. MSN-NH_2_ alone was nontoxic to melanoma cells when incubated for 48 h in the range of 0 to 468 µg/mL. The combination of 5-FU MSN-NH_2_ and DEX MSN-NH_2_ showed significant increase in toxicity compared to their free dug counterparts and exhibited a synergetic effect as well as the ability to circumvent DEX induced 5-FU resistance in melanoma cells.

## 1. Introduction

Malignant melanoma is among the deadliest types of skin cancer and comprises the majority of cases [1,2]. The incidence of skin cancer is on the rise. It is estimated that in 2020 a minimum of 100,000 new cases will be diagnosed and 6000 new deaths will occur in the USA alone [3]. The management of melanoma with systemic therapy, such as chemo- or radiotherapy, is associated with a high rate of resistance as well as significant side effects and toxicities. Consequently, a possible treatment failure among many patients can be predicted [4,5]. The high drug resistance rates detected with the current treatments of melanoma urge the investigation of new treatment approaches. For this purpose, many researchers have proposed the utilization of a combined drugs therapy [6,7]. Others have even proposed the concurrent delivery of two or more agents by the same delivery system for a maximum benefit [7,8].

Since 2001, mesoporous silica nanoparticles (MSN) have been utilized as drug carriers in various applications [9,10]. Some advantages of MSN over other drug delivery systems is their tunable particle size ranging between 50 nm and 300 nm, large surface area rendering them high loading capacity, and two functional surfaces that can be selectively functionalized or conjugated with targeting ligands [11,12]. Their superiority as transdermal drug delivery vehicles is due to their ability to provide a targeted therapy, improved drug pharmacokinetics, improved drug efficacy, and reduced side effects [9,13]. Previous reports have shown that MSN can adsorb or encapsulate relatively large amounts of drug molecules while exhibiting well-known physicochemical stability providing them with high resistance to heat, pH, mechanical stress, and degradation by hydrolysis [14,15]. Although the diameter of MSN is critical for cellular uptake, proper MSN surface characteristic is also important in achieving high drug loading, leading to the release of high drug concentration at the site of interest [11]. In fact, adding an amine group on the surface of MSN results in the formation of amino-functionalized mesoporous silica nanoparticles (MSN-NH_2_), which could further improve the encapsulation of drugs through hydrogen bonding or electrostatic interactions [16,17].

The functionalization process is traditionally performed through post-synthesis grafting [16,18]. This approach can lead to an uncontrollable distribution of amino groups on the surface and presence of amine groups at the entrance of the mesochannels, consequently reducing the number of accessible pores negatively impacting the textural properties of the MSN and the efficiency of drug encapsulation [19]. On the other hand, the in situ co-condensation process of amino functionalization showed a more homogeneous distribution of amino groups on the silica framework. The presence of amino groups within silica walls is inevitable with this method of functionalization [20,21].

In this work, we describe the synthesis and characterization of MSN-NH_2_ nanoparticles prepared in situ utilizing an anionic surfactant and a co-structure directing agent (CSDA), 3-aminopropyltriethoxysilane (APTES). Furthermore, we evaluated the potential impact of changing the volume of APTES on the structure and texture of the nanoparticles. Subsequently, we investigated the loading capacity and release profile of 5-Fluorouracil (5-FU) and Dexamethasone (DEX) from MSN-NH_2_. Finally, we evaluated the topical efficacy of 5-FU MSN-NH_2_ and DEX MSN-NH_2_ by studying the permeation and accumulation through the skin, as well as the cytotoxicity against melanoma cells.

## 2. Materials and Methods

### 2.1. Materials

*N*-lauroylsarcosine sodium, 3-aminopropyltriethoxysilane (APTES), tetraethyl orthosilicate (TEOS), ammonium acetate, 5-fluorouracil, and dexamethasone were purchased from Sigma-Aldrich Chemical Co. (St Louis, MO, USA). Carbopol 934 was purchased from Acros Organics (Morris Plains, NJ, USA). All other chemical reagents used were of analytical grade and used without further purification. HT-144 (ATCC^®^ HTB-63™), and McCoy’s 5a Medium Modified (ATCC^®^ 30-2007™) was obtained from American Type Culture Collection (ATCC, Manassas, VA, USA).

### 2.2. Synthesis of MSN-NH_2_

To synthesize MSN-NH_2_, 1.4667 g (1 mmol) of N-lauroylsarcosine sodium was dissolved in 33 mL water:ethanol mixture (10:1), then 4 mL 0.1 M HCl was added with stirring for 1 h. Thereafter, different volumes of APTES (50, 75, 100, and 150 mL) were added to the mixture and stirred for 10 min. TEOS (1.5 mL) was then added to the reaction mixture and stirred for 10 min. The mixture was subjected to ultrasonic waves that were produced using an ultrasonic water bath (Cole-Parmer SS, Cole-Parmer, Vernon Hills, IL, USA).

The mixture was left to rest for 1 h, it was then heated at 80 °C for 18 h. The final solid product was recovered by centrifugation at 10,000 rpm, washed with deionized water, and dried in an oven at 60 °C for 12 h. To maintain the presence of amino functional group within mesochannels, surfactant molecules were removed by solvent extraction method where the obtained powder was dispersed in ammonium acetate (8.01 g) in 100 mL (4:1 ethanol:H_2_O) and refluxed at 90 °C for 12 h [22].

### 2.3. Characterization of the MSN-NH_2_

Transmission electron microscopy (TEM) images were obtained using a JEM-2100F electron microscope (JEOL, Tokyo, Japan) operated at 200 kV. Nitrogen (N_2_) sorption isotherms were measured at 77 K with NOVA 4200e analyzer (Quantachrome, Boynton Beach, FL, USA). Before measurements, samples were degassed in vacuum at 200 °C for at least 18 h. The Brunauer–Emmett–Teller (BET) method was utilized to calculate the specific surface area using adsorption data at a relative pressure range of 0.02 to 0.20. By using the Barrett–Joyner–Halenda (BJH) model, pores volume (Vt) and size distributions were derived from the adsorption branches of isotherms. Total Vt was estimated from the adsorbed amount of nitrogen molecules at a relative pressure P/P_0_ of 0.995. The Fourier transform infrared (FT-IR) spectra were recorded using the Vertex-80/80V spectrometer (Bruker, Billerica, MA, USA).

### 2.4. Preparation of 5-FU or DEX (5-FU/DEX) MSN-NH_2_

5-FU/DEX were loaded into MSN-NH_2_ using the wet impregnation method [17]. To prepare 5-FU MSN-NH_2_, 5-FU was dissolved in 25 mL of PBS (pH 7.4) to make 1 mg/mL solution. To prepare a 5-FU:MSN-NH_2_ in a 1:1 ratio (*w*/*w*), 25 mg MSN-NH_2_ was added to the 5-FU in PBS solution. The amounts of 5-FU and MSN-NH_2_ were adjusted accordingly to achieve 5:1 (*w*/*w*) ratio. Mixtures were then sonicated for 5 min by probe sonication (Badnelin, Germany), then stirred at room temperature for 3 h while the evaporation of the solvent was prevented. The suspension was centrifuged at 6000 rpm for 1 h to separate the loaded MSN-NH_2_, then dried under a vacuum oven at 60 °C. The dried powder was dispersed in PBS (pH 7.4), stirred for few seconds, centrifuged, and dried under vacuum.

DEX MSN-NH_2_ was prepared in 1:1, 1:5, and 5:1 ratios (*w*/*w*) using the same method described above, except for DEX was dissolved in ethanol instead of PBS before the addition of MSN-NH_2_. Finally, 5-FU MSN-NH_2_ and DEX MSN-NH_2_ were mixed at a 1:1 ratio under stirring for 2 h to obtain 5-FU MSN-NH_2_ + DEX MSN-NH_2_ drug combination. The loaded drugs in MSN-NH_2_ were extracted using PBS (pH 7.4) and the amount of drugs loaded was determined using HPLC (Agilent, Santa Clara, CA, USA) and UPLC (Waters, Milford, MA, USA) for 5-FU and DEX, respectively [23,24]. Encapsulation efficiency (EE%) and loading capacity (LC%) were calculated according to the following equations,

(1) $$\mathrm{EE}\%\;=\;\frac{W_{\mathrm{total}\;}\;{-W}_{\mathrm{free}}}{W_{\mathrm{total}\;}}\;\times \;100$$

(2) $$\;\mathrm{LC}\%\;=\;\frac{W_{\mathrm{total}\;}{-W}_{\mathrm{free}}}{w}\;\times \;100\;$$

where *W*_total_ is the initial weight of the drug before loading, W_free_ is the excess amount of drug in the solution, and W is the total weight of the drug and MSN-NH_2_.

### 2.5. Characterization of 5-FU/DEX MSN-NH_2_

The morphology of MSN-NH_2_ and particle size were determined by transmission electron microscope (TEM) (JEM-1011, JEOL, Tokyo, Japan) at 60 kV. The stability profile of MSN-NH2 in suspension was performed using the previously reported method with minor modification [25]. Dried MSN-NH2 was weighed (0.25 mg/mL) and suspended in distilled water using a round shaped bottle and placed in shaking water bath (100 rpm) at 37 °C for 48 h. At different time intervals, the particle size was analyzed using Zetasizer (Nano ZS, Malvern, UK). The particle size of MSN-NH2 at different time points helped to monitor any aggregation as an indicator of MSN-NH2 stability.

FT-IR spectra of 5-FU, DEX, and MSN-NH_2_ were recorded on a spectrum 100 (PerkinElmer Waltham, Waltham, MA, USA) using potassium bromide (KBr) disc technique and scanned over the range of 5000 to 400 cm^-1^. The thermal behavior of the samples was conducted using a differential scanning calorimetry (DCS) DSC-8000 (Perkin Elmer Instruments, Waltham, MA, USA) at a scan rate of 10 °C/min covering the temperature range of 25–350 °C and the zeta-potential was calculated by electrophoretic measurements using the Zetasizer Nano ZS (Malvern Instruments, Malvern, UK) at 25 °C.

### 2.6. In Vitro Drug Release Study

To facilitate a localized easy topical (skin) application, Carbopol was used as a topical vehicle for 5-FU MSN-NH_2_ + DEX MSN-NH_2_ as well as for free drugs. A weighed amount of Carbopol 934 was dispersed in distilled water (1% *w*/*v*). Dispersion was stirred for 3 h at room temperature, and the pH was adjusted to 7.4 using triethanolamine (0.5% *w*/*v*). The gel base was then allowed to stand overnight to enable any trapped air to escape and to allow the cross-linking between Carbopol 934 and triethanolamine to take place. An appropriate amount free 5-FU and DEX free or loaded into MSN-NH_2_ were incorporated into the gel to a final concentration of 0.08% *w*/*w* for 5-FU and 0.2% *w/w* for DEX. The obtained formulations were stirred at room temperature until Carbopol gels were homogeneous.

The release of 5-FU MSN-NH_2_ + DEX MSN-NH_2_-gel was performed using modified Vertical Franz diffusion cells. The cellulose membrane molecular weight cut-off of 14,000 (Sigma Co., St. Louis, MO, USA) was used after an overnight immersion in PBS (pH 7.4). The receptor phase consisted of 25 mL of 20% ethanol in PBS (pH 7.4) solution and was maintained at 37 °C with constant stirring. The obtained gel of about 0.5 g incorporating free drug or 5-5-FU MSN-NH_2_ + DEX MSN-NH_2_ was applied to the cellulose membrane and carefully spread to achieve complete uniform coverage. The donor compartment was exposed to PBS for 24 h. At predetermined time intervals, 1 mL of the release media was withdrawn, and an equivalent volume of PBS maintained at 37 °C was added to the receiver compartment to maintain sink condition. Samples were then analyzed using HPLC and UPLC to determine the release of 5-FU and DEX, respectively, and compared to the release profiles of free drug gel.

To study the release mechanism from the different type of MSN-NH2, five different kinetic models were considered to fit the experimental data. Drug release parameters were calculated by the following mathematical models; zero-order, first-order, Higuchi, and Korsmeyer–Peppas. Release data were fitted into the model equations to identify the release mechanism of drugs from formulations:

Zero-order equation: Q_t_ = Q_0_/K_0_t (3)

First-order equation: logQ_t_ = logQ_0_/K_1_t/2:303 (4)

Higuchi equation: Qt = K_h_t^1/2^ (5)

Korsmeyer-Peppas equation: Q_t_/Q_∞_ = K_p_t*^n^* (6)

where Q_t_, Q_0_, and Q_∞_ represent the cumulative amount of drug released at time t, initial amount of drug, and total amount of drug in dosage form, respectively. K_0_ is the zero-order release rate constant obtained by plotting Q_t_ against time. K_1_ represents the first-order release rate constant determined by plotting log(Q_t_/Q_∞_) against time. K_h_ is the Higuchi release rate constant obtained by plotting Q_t_ against the square root of time. K_p_ denotes the release rate constant of the Korsmeyer–Peppas model, and the constant *n* is the release exponent which is used for characterizing the different release mechanisms.

The parameters K_p_ and n can be calculated by plotting log(Q_t_/Q_∞_) against log of time.

All the above mathematical models are only valid for the first 60% of the drug released from the MPs. Experimental data were analyzed by nonlinear least regression, using Origin Pro 8 software.

To differentiate the drug release mechanism, we further used the Peppas–Sahlin model as shown in Equation (7).

F = 1/(1 + (K2/K1) × t*^n^*) (7)

In this model, the right-hand side is the contribution of the Fickian diffusion and the rest is the is related to polymer relaxation (Peppas and Sahlin) where K_1_ and K_2_ are kinetic constants, and *n* is the diffusion exponent. The fraction of drug release F due to the Fickian diffusion mechanism can be calculated according to Equation (4).

### 2.7. Ex Vivo Skin Permeation Study

The efficiency of a transdermal formulation is determined by the amount of penetrant and extent of permeability at the site of administration. Therefore, an ex vivo skin permeability study of 5-FU/DEX MSN-NH_2_-gel was performed in comparison to free drug in solution or in gel.

Eighteen male Wistar rats weighing 200–250 g were obtained from the animal house, College of Pharmacy, King Saud University, Riyadh, Saudi Arabia. All animals were treated following protocol and guidelines of the Ethical Committee for Performing Studies on Animals, King Saud University, Riyadh, Saudi Arabia, and protocol number SE-19-151, approved at 13-02-2020. Animals were divided into 6 groups (*n* = 3). Rats were euthanized with a ketamine HCl and xylazine mixture (Ketamine HCl 100 mg/kg and Xylazine 10 mg/kg). The skin preparation was performed as described previously [26]. Prepared skin samples were mounted onto Franz diffusion cells (Logan Instrument Corp., Somerset, NJ, USA) with a diffusion area of 1.7 cm^2^ and a receptor volume of about 12 mL. The receptor fluid (20% ethanolic solution in PBS pH 7.4) was kept at 32 ± 1 °C (skin temperature) throughout the experiments and stirred at 500 rpm using a magnetic stirring (Sigma-Aldrich, Taufkirchen, Germany) to mimic in vivo conditions. An appropriate amount of each formulation (5-FU solution, 5-FU gel, 5-FU MSN-NH_2-_gel, DEX solution, DEX gel, or DEX MSN-NH_2-_gel) was applied to the skin surface and carefully spread to achieve complete uniform coverage. All experiments were carried out with occluded donor compartments. One milliliter of the sample was withdrawn at predetermined time intervals, and the same volume of fresh medium was replaced to maintain sink conditions. The effective diffusion area of the skin sample was carefully wiped-off using fresh receptor solution in triplicate. Then, the skin was stripped to separate stratum corneum (SC) using 10 tapes [26]. Subsequently, the remaining skin was cut into small pieces. The amount of the drug in the tapes and skin pieces was extracted using 75% acetonitrile solution under vortexing followed by ultrasonic (Cole-Parmer SS, Cole-Parmer, Vernon Hills, IL, USA) extraction for 30 min. Extracts, as well as receptor fluid were filtered, and drug concentration was determined as described above. The efficiency of the extraction was confirmed by spiking a known amount of the drug into tapes and the remaining skin.

Permeation profiles were constructed by plotting the cumulative amount of drug (µg) permeated per unit skin area (cm^2^) versus time (t). The drug flux (Jss), permeation rate as μg/cm^2^.h, through the rat skin was calculated by dividing the slope of the linear portion of the graph with the area of the diffusion cell. The extrapolation of the linear part of the curve to intercept with the X-axis was equal to lag time (T_L_). The permeability coefficients (Kp, cm^2^/h) were obtained through dividing the Jss by initial drug concentration (C_0_) in the donor compartment. To determine the extent of penetration enhancement, an enhancement ratio (ER) was calculated by dividing the Jss of respective formulation with Jss of free drug in solution [27].

### 2.8. In Vitro Cytotoxicity Assay

The cytotoxicity of 5-FU/DEX MSN-NH_2_ and 5-FU MSN-NH_2_ + DEX MSN-NH_2_ were evaluated in melanoma cells (HT-144). Cells were maintained at 37 °C, 5% CO_2_ in McCoy′s 5a Modified Medium with 10% FBS. To perform the cytotoxicity assay, cells were seeded in a 96-well culture plate at a density of 1 × 10^4^/well in 100 µL culture media and incubated for 24 h. For 5-FU MSN-NH_2_, cells were treated with a serial dilution of 5-FU ranging from 0 to 50 μg/mL with wells treated with an equivalent amount of free MSN-NH_2_ or free-5-FU used as controls. On another set of plates, cells were treated with DEX MSN-NH_2_, with DEX concentrations ranging from 0 to 125 μg/mL. Cells treated with corresponding amounts of drug free MSN-NH_2_ or free DEX served as controls. The last set of cells were treated with a drug combination (5-FU MSN-NH2 + DEX MSN-NH2) maintaining the drug ranges described above. Cells treated with corresponding amounts of drug free MSN-NH_2_ and free 5-FU + DEX drug combination served as controls. Cells were then incubated for 24 and 48 h, 20 μL of 2.5 mg/mL of 3-(4,5-dimethylthiazol-2-yl)-2,5-diphenyltetrazolium bromide (MTT) in PBS was added to cells and cell were further incubated for 4 h at 37 °C. MTT solution was then completely removed and 100 μL DMSO was added to solubilize formazan crystals. Absorbance was measured at 540 nm using Spectramax 250 microplate reader (Molecular device, San Jose, CA, USA). Cell viability (%) was calculated as [optical density (OD) of treated cells/OD of control cells] × 100 [28].

### 2.9. Statistical Data Analysis

Statistical analysis and plotting graphs were performed using GraphPad Prism software (GraphPad Software Inc., San Diego, CA, USA). All experiments were conducted in triplicate. Results are expressed as mean ± SED, and *p* < 0.05 was considered significant. Analysis of variance (ANOVA) and Tukey′s multiple comparisons test were used when comparing three or more groups.

## 3. Results and Discussion

### 3.1. Synthesis and Characterization of MSN-NH_2_

**Figure 1:**
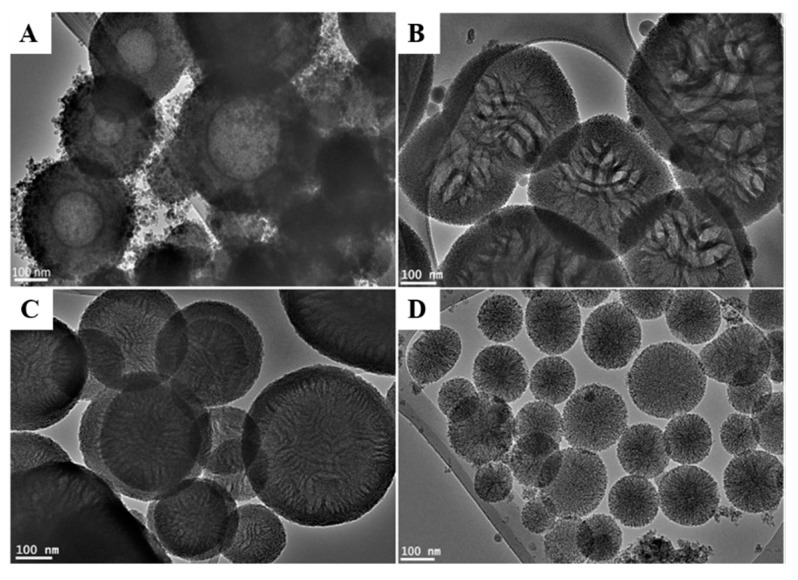
TEM images of MSN-NH_2_ prepared with different volumes of 3-aminopropyltriethoxysilane (APTES): (**A**) 50 μL, (**B**) 75 μL, (**C**) 100 μL, and (**D**) 150 μL. Scale bar = 100 nm.

In this study, we demonstrated that functionalization of mesoporous silica spheres with amin groups can be accomplished through the utilization of an anionic surfactant (*N*-lauroylsarcosine sodium) with a co-structure directing agent (CSDA), such as 3-aminopropyltriethoxysilane (APTES), and a silica precursor. Herein, negatively charged anionic surfactant molecules (S^−^) can interact with negatively charges silica species (I^−^) through the mediation of APTES (N^+^) via S^−^N^+^I^−^ pathway providing mesoporous silica with highly distributed amino functional groups and superior activity [29]. To understand the impact of utilizing different volumes of APTES on the morphology of MSN-NH_2_, TEM observation was conducted and is shown in Figure 1. Low APTES volumes (50 μL) resulted in the formation of hollow silica spheres with different particle sizes ranging from 300 to 450 nm and a thick shell of 50–70 nm (Figure 1a). Increasing APTES to 75 μL resulted in a remarkable change of the external and internal morphology of the formed particles, and the formation of cubic like mesoporous particles with a size range of 300 to 460 nm with a mesoporous shell of 33–70 nm in thickness. Interestingly, the internal structure of the particles showed a leaf-like interior with large cavities and a lamellar matrix (Figure 1b). To obtain silica spheres with a mesoporous interior, we opted to increase the volume of APTES used. A further increase in APTES to 100 μL gave silica spheres that were 200–450 nm in size with a thinner shell of 35–50 nm in thickness (Figure 1c). However, particles still showed a leaf-like interior, but with a much smaller cavity compared to the particles synthesized by 75 μL of APTES. Finally, 150 μL of APTES yielded mesoporous silica spheres that are 90–230 nm in size (Figure 1d).

The difference in structural properties of the formed silica spheres can be attributed to the change in diffusivity and reactivity of APTES as the volume increases. When mixing water and ethanol, an emulsion like mixture is formed induced by the high dielectric constant of water compared to ethanol [30]. The ethanol droplet is then stabilized by anionic surfactant molecules concentrating at the hydrophobic region (forming the core), whereas water will mainly concentrate at the hydrophilic region of the anionic surfactant micelle [30]. APTES mediates the electrostatic interaction between the anionic surfactant and hydrolyzed silica species which will construct a mesoporous shell at 50 μL. Increasing the volume to 75 μL allows APTES to diffuse deeper into and disrupt the ethanol droplet (hollow cavity) while reacting at different sites of the core pulling along surfactant and silica species, and replacing the hollow cavity of the sphere with a leaf-like interior. APTES also continues to interact at the periphery of the silica spheres forming a mesoporous shell. At 100 μL, the diffusivity of APTES into ethanol increases encouraging more surfactant and silica to move to the center of the sphere, reducing the size of and transforming the leaf-like interior into a more organized mesoporous matrix (Figure 1c). Finally, at 150 μL APTES facilitates a better mixing of ethanol and water forcing the emulsion droplet to disappear and form a conventional mesoporous silica sphere.

**Figure 2:**
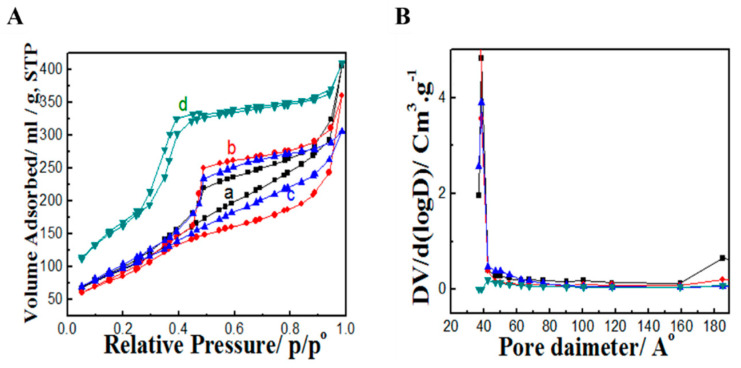
(**A**) N_2_ sorption isotherm and (**B**) pore size distribution of MSN-NH_2_ prepared with different volumes of APTES: (a) 50 μL, (b) 75 μL, (c) 100 μL, and (d) 150 μL.

To further characterize MSN–NH_2_ nanoparticles, N_2_ sorption analysis was performed and is shown in Figure 2A. All samples possessed type IV adsorption/desorption isotherm which is characteristic of mesoporous materials. Samples prepared with 50–100 μL of APTES showed a distinct hysteresis loop that extended to a wide range of relative pressure indicating a large inner cavity or hollow structure. On the other hand, the mesoporous silica sample prepared with 150 μL of APTES did not show such a large hysteresis loop, suggesting the absence of a hollow cavity. These results are consistent with TEM results described earlier. BET surface area-pore volume textural properties were 368.19 m^2^/g–0.628 cc/g, 345.78 m^2^/g–0.557 cc/g, 373.26 m²/g–0.473 cc/g, and 619.14 m²/g–0.633 cc/g for 50, 75, 100, and 150 μL APTES, respectively. These results suggest that despite mesoporous silica spheres prepared with 150 mL, they did not possess a hollow cavity or a leaf-like interior, they exhibited superior textural properties that may in turn affect drug encapsulation efficiency. It is noteworthy to mention that changing the volume of APTES did not affect the size of the mesopores as shown by the pore size distribution of MSN-NH_2_ presented in Figure 2B where all samples had mesochannels of about 4 nm in diameter.

**Figure 3:**
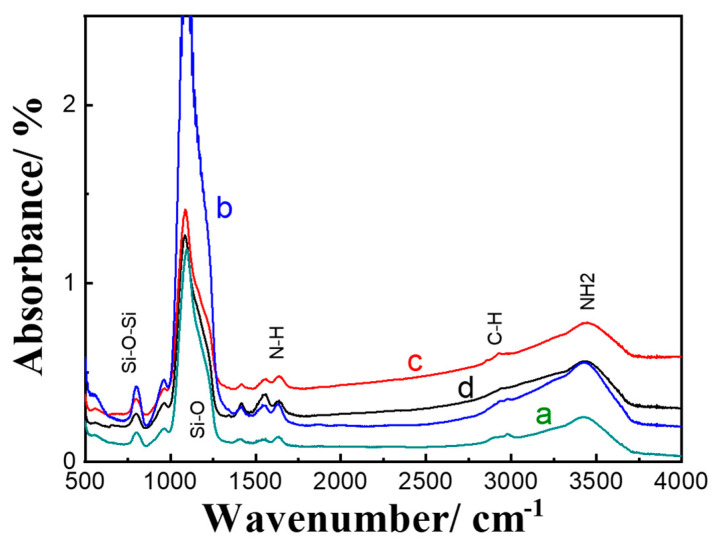
FTIR spectra of MSN-NH_2_ prepared with different volumes of APTES: (a) 50 μL, (b) 75 μL, (c) 100 μL, and (d) 150 μL.

To confirm the amino functionality of the mesoporous silica spheres, FTIR measurements were conducted. Figure 3 shows Si–O bending and Si–O–Si asymmetric stretching noticeable at 560 and 1150 cm^−1^, respectively, which are characteristic for the silica group. The silanol group (Si–OH) can be noticed as a weak peak found at around 950 cm^−1^. The N–H groups of the mesoporous silica synthesized with different volumes of APTES appeared at 1250 to 1750 cm^−1^. Peaks characteristics of CN stretching and NH bending can be found between 1229 and 1301 cm^-1^ and 1480 and 1575 cm^−1^, respectively [31]. The broadband at 2700–3500 cm^−1^ can be attributed to the aminopropyl groups were N–H stretching at 3346 cm^−1^ is characteristic of free amine along with terminal amino groups at around 3305 cm^−1^ [32]. The gradual increase in the intensity of N–H band at 1300–1700 cm^−1^ can be attributed to the increment of the amino content in the formed spheres in correlation with increasing the volume of APTES. Collectively, MSN-NH_2_ synthesized with 150 μL APTES showed optimal characteristics and thus was utilized for further evaluation.

### 3.2. Preparation and Characterization of 5-FU/DEX Loaded MSN-NH_2_

Designing an optimal transdermal drug delivery system to target melanoma requires the utilization of effective drugs that could exert the needed anticancer effect once reaching cancer cells. 5-FU is an anticancer drug that belongs to the antimetabolite class, and represents one of the major anticancer drugs used topically to treat premalignant and malignant conditions of the skin, such as Bowen’s disease and superficial basal cell carcinomas [33,34]. Despite proving its safety and efficacy following topical application in management of skin conditions, transdermal permeation of 5-FU through the lipophilic stratum corneum remains very challenging owing to the high polarity of 5-FU (log P octanol/water = −0.89) and hydrophilicity [15,35,36,37]. This has prompted several investigators to explore alternate approaches to facilitate the delivery of 5-FU across the skin. DEX, on the other hand, has been successfully used concurrently with anticancer agents because of its anti-inflammatory and immunosuppressive effects, which reduce some of the side effects associated with anticancer agents such as nausea and vomiting [38]. Additionally, a recent study has demonstrated that DEX can induce apoptosis in melanoma cells when taken in high doses [38]. However, a number of side effects associated with the systemic use of DEX, such as hypertension, hydro-electrolytic disorders, hyperglycemia, peptic ulcers, and glycosuria, restricts the use of DEX for prolonged therapy [39]. Therefore, we investigated the potential use of MSN-NH_2_ as transdermal drug delivery carriers for 5-FU and DEX.

**Figure 4:**
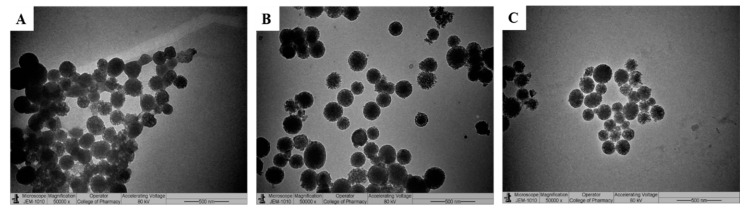
TEM images of MSN-NH_2_ after drug loading. DEX MSN-NH_2_ (**A**), 5-FU MSN-NH_2_ (**B**), and combined MSN-NH_2_; mixed DEX MSN-NH_2_ and 5-FU MSN-NH_2_ (1:1 ratio) (**C**). bar = 500 nm.

**Figure 5:**
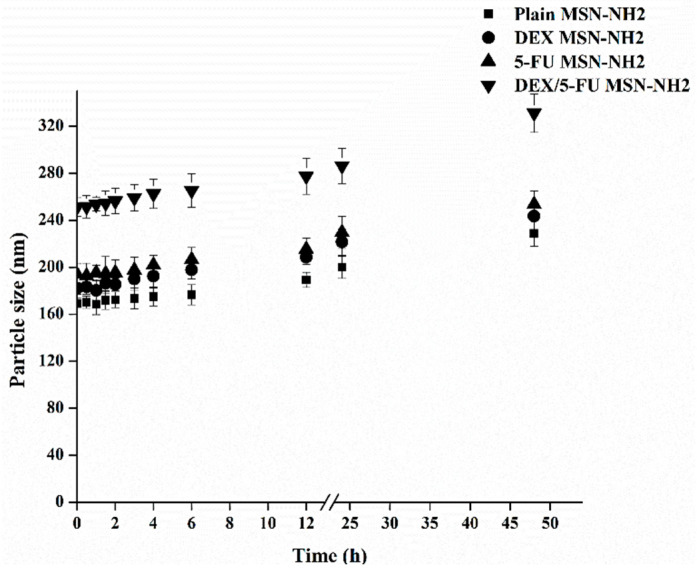
Particle size of free and drug loaded MSN-NH_2_ measured by DLS in distilled water as a function of time.

To obtain optimal drug loading, different drugs to MSN-NH_2_ ratios were investigated. The effect of changing drugs to MSN-NH_2_ ratio on EE% and LC% are presented in Table 1. The LC% of DEX increased from 9.5% to 44.72% as the ratio changed from 1:1 to 5:1 (DEX: MSN-NH_2_). This could be due to the presence of high interaction between DEX (anionic drug) and the cationic surface of MSN-NH_2_ [40]. On the other hand, LC% of 5-FU was not affected by manipulating 5-FU to MSN-NH_2_ ratio as no significant difference was found when using 1:1 or 5:1 5-FU to MSN-NH_2_ (Table 1). Therefore, the ratios of 1:1 for 5-FU: MSN-NH2 and 1:5 for DEX:MSN-NH2 were used for the rest of the studies. Although previous reports indicated that MSN-NH_2_ can increase the loading capacity of 5-FU due to the electrostatic interactions between the negatively charged 5-FU and positively charged amino groups, loading can also be affected by the degree as well as the distribution of functional groups on the MSN surface [17]. Furthermore, at pH 7.4, only 25% of 5-FU (pKa 8) is ionized, therefore, we do not expect that much electrostatic interaction occurred between 5-FU and the surface amin group [41]. Thus, 5-FU drug loading might be mainly through MSN-NH_2_ surface coating and pores entrapment [42]. Following drug loading, TEM images of MSN-NH_2_ (Figure 4) were taken and they showed that all MSN-NH_2_ was distributed homogeneously (Figure S1B,C). All investigated MSN-NH_2_ dispersions showed particles size in the nano-range (169.3 ± 4.2 nm) with exceptionally low PDI (0.057). The sizes of 5-FU MSN-NH_2_ and DEX MSN-NH_2_ were 193.9 ± 8.7 nm (0.219) and 188.2 ± 5.4 nm (179), respectively. The increase in particle size could be attributed to the loading of the drugs. The recorded values of PDI represented a relatively monodispersed particle size distribution. The loaded MSN-NH_2_ had a diameter slightly higher than the empty MSN-NH_2_, thus indicating a partial deposition of the drug on MSN-NH_2_ surface. As it is crucial to preserve the stability of MSN-NH2 in order to maintain pharmacological and cytotoxic activities of the encapsulated drugs, the stability of the MSN-NH2 was investigated at various time intervals by measuring the size distribution (Figure 5). Results indicated that there was no visible aggregation/sedimentation of the particles. Drug-free or loaded MSN-NH2 showed good stability to up to 6 h with a slight increase in particle size at 12 h. Results also indicated that the size of the nanoparticles moderately increased over 48 h. Results suggested that MSN-NH2 had good stability, probably due to the presence of amino groups on the surface of the nanoparticles [25].

**Figure 6:**
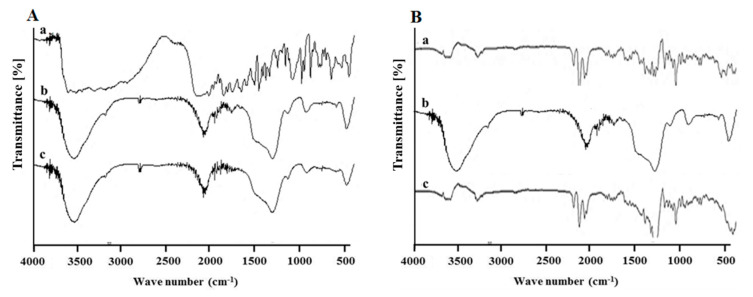
(**A**) FT-IR spectra of (a) 5-FU, (b) MSN-NH_2_, and (c) 5-FU MSN-NH_2._ (**B**) FT-IR spectra of (a) DEX, (b) MSN-NH_2_, and (c) DEX MSN-NH_2._

**Figure 7:**
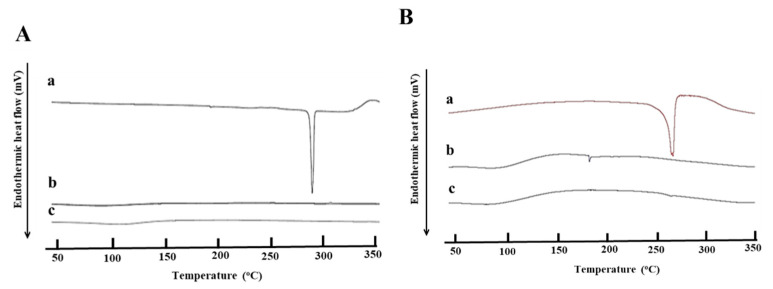
(**A**) DSC Thermograms of (a) 5-FU, (b) MSN-NH_2_, and (c) 5-FU MSN-NH_2_. (**B**) DSC thermograms of (a) DEX, (b) MSN-NH_2_, and (c) DEX MSN-NH_2._

Next, FTIR analysis was performed to investigate if there was a chemical interaction between drugs and MSN-NH_2_. The intense peaks shown in the FTIR spectra (Figure 6A) at 3160, 1727, 1662, 1426, 1247, 811.7, and 547 cm^−1^ are due to the stretching vibration of amide (amide II and amide III) and the aromatic ring in the structure of 5-FU. Following drug loading, most of the intense characteristic peaks of 5-FU were not observed in the same positions, suggesting a possible interaction between 5-FU and MSN-NH_2_ and confirming that 5-FU was encapsulated in the MSNs [43,44]. On the other hand, DEX peaks at 1662 cm^−1^ (CO vibration) and 1610 cm^−1^ (C=C vibration) and MSN-NH_2_ peaks, which were retained after drug loading, suggest the absence of chemical interaction between the drug and MSN-NH_2_. This confirms that DEX loading was mainly due to a simple surface adsorption (Figure 6B) [17,45]. Drug loading into MSN-NH_2_ was also confirmed by the DSC analysis. The thermogram in Figure 7A(a) shows that 5-FU DSC curve exhibited a single endothermic peak at 285 °C, which corresponds to the intrinsic melting points of 5-FU. However, no drug melting peak was identified with DSC curves after drug loading (Figure 7A(c)). A similar scenario was found with DEX DSC curve showing a single endothermic peak at 272.6 °C, which corresponds to DEX intrinsic melting points (Figure 7B(a)), and no melting peak was identified after DEX loading in MSN-NH_2_ (Figure 7B(c)). It is noteworthy to mention that co-loading both drugs in the same MSN-NH2 batch was difficult to achieve since it was challenging to dissolve both drugs in a one common solvent. Instead each drug was loaded into MSN-NH_2_ and purified separately. Finally, a weighed amount of each drug loaded MSN-NH_2_ were mixed dispersed in Carbopol gel. In light of the above findings, results suggest that drugs are in a nanocrystalline state and that drugs are mostly encapsulated within the MSN-NH_2._

### 3.3. In Vitro Release Studies

**Figure 8:**
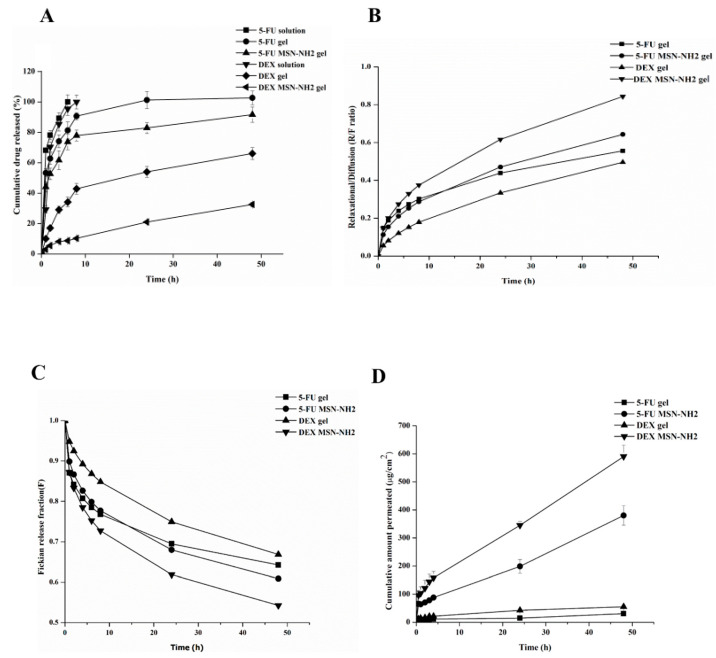
Release profiles of 5-FU and DEX in PBS (pH 7.8) from MSN-NH_2,_ free in gel, and control (**A**). Relaxational to diffusional ratio as a function of time for DEX and 5-FU from MSN-NH2 at pH of 7.4 (**B**). The Fickian release fraction of DEX/5-FU MSN-NH_2_ gel (**C**). Permeability of 5-FU and DEX in MSN-NH_2_ and free in gel (**D**). Data are represented as mean ± SD (*n* = 3).

To facilitate dermal application, Carbopol 935 gel was utilized in these studies as a topical delivery vehicle for 5-FU MSN-NH_2_ + DEX MSN-NH_2_ as well as free drugs. A gel formula was selected for obvious reasons such as easy skin application and wash-off, suitability for application on hairy skin areas, adoptability to the shape of skin, as well as being aesthetically appealing to many patients [46,47]. The in vitro release profiles of 5-FU MSN-NH_2_ + DEX MSN-NH_2_-gel are represented in Figure 8A. Results indicate that the release of 5-FU and DEX from MSN-NH_2_-gel follow a controlled release pattern compared to their free drugs counterparts. This favorable release pattern of drugs from the nanoparticle system, for 5-FU in particular, has proven to be beneficial in prolonging the time of drug action and overcoming drug resistance as well as reducing any potential side effects [48,49]. Release profiles can further confirm the presence of an interaction between 5-FU MSN-NH_2_ + DEX MSN-NH_2_. However, the difference in release profiles between the two drugs, cumulative released at 24 h was 83% and 21% for 5-FU and DEX, respectively, could be attributed to the difference in the physicochemical characteristics of drugs, namely, differences in hydrophilicity. Therefore, 5-FU was released rapidly from MSN-NH_2_-gel compared to DEX. The initial burst release of 5-FU at pH 7.4, which was around 58% during the first two hours, suggests that 5-FU adsorbed on the surface and at pores entrance of MSN-NH_2_ was released first followed by a slow release of drug entrapped within MSN pores or electrostatically attracted to the NH_2_ [50]. On the other hand, the slower release of DEX from MSN-NH_2-_gel compared to 5-FU can be due to the strong interaction between MSN-NH_2_ and DEX (34). The cumulative released percentage of DEX form MSN-NH_2_ was 10% at 8 h and 33% at 48 h compared to around 79% and 92% of 5-FU at 8 h and 48 h, respectively. Data provided from the drug release study was fitted into different release kinetics models, i.e., zero-order, first-order, Higuchi, and Korsmeyer–Peppas models (Table 2). Plotted data showed that the highest regression coefficient (R^2^) was in agreement with Korsmeyer–Peppas and Higuchi models indicating that drug release from the gel system, either loaded in MSN-NH_2_ or in a free form was mainly through diffusion followed by relaxation of the MSN-NH_2_-gel [50].

The Peppas–Sahlin model indicates that diffusion prevailed with MSN-NH_2_. This model also showed the high diffusion and release rate of 5-FU from the gel only and MSN-NH_2_ gel. The obtained values of diffusional exponent (m) for MSN-NH_2_ was used to reveal the mechanism of the controlled drug release. The ratio of the relaxational to the diffusional behavior (R/F) of MSN-NH2 is depicted in Figure 8B. The R/F ratio increased over time, indicating that relaxation/gelling contribution of the MSN-NH_2_ gel was the complementary process of drug diffusion.

Nonlinear regression analysis was performed on curves obtained from data fitted into the Peppas–Sahlin model to obtain rate constants, which can indicate the mechanism of drug release from MSN-NH2 gel. Correlated results of controlled drug release data fitted in Peppas–Sahlin model are shown in Table 3. *R*^2^ values of data fitting were >0.99. This revealed that the in vitro release data of the MSN-NH_2_ could be successfully fitted using the Peppas–Sahlin model. The *F* values for Fickian diffusion were calculated using Equation (7) and are presented in Figure 8C. As found with drug loaded in MSN-NH_2_ gel, the impact of the release mechanism related to relaxation of the polymer increased with respect to the Fickian mechanism.

### 3.4. Ex Vivo Skin Permeability Studies

The ex vivo permeation of 5-FU/DEX was tested in rat skin using Franz diffusion cells. Permeation data of 5-FU/DEX MSN-NH_2_ gel were compared to the corresponding free drug gel (control) for up to 48 h. The permeation profiles of 5-FU/DEX from MSN-NH_2_ or free in gel through the skin are shown in Figure 8C. MSN-NH_2_ gel showed higher permeation into the skin compared to control in the receptor media after 48 h (380.2 µg/cm^2^ of 5-FU MSN-NH_2-_gel vs. 30.9 µg/cm^2^ of free 5-FU-gel and 590.4 µg/cm^2^ of DEX MNS-NH_2-_gel vs. and 55.5 µg/cm^2^ of free DEX-gel). However, DEX exhibited higher skin permeability in comparison to 5-FU [51]. The lower skin permeability of 5-FU compared to DEX could be attributed to the barrier properties of the stratum corneum and the aqueous solubility of 5-FU [52,53]. With MSN-NH_2_, a relative burst in the permeability of DEX and 5-FU was observed in the first 4 h, after which the rate of drug permeability was sustained to up to 48 h. Table 4 summarizes drug permeability parameters of the ex vivo diffusion study. All formulations exhibited a short lag time of 0.5 h. Results confirmed that DEX MSN-NH_2_ and 5-FU MSN-NH_2_ retained an optimized skin permeability showing significantly higher drug flux and permeability coefficient than controls (*p* < 0.05). The enhancement ratios of 5-FU MSN-NH_2_ gel to free drug and DEX MSN-NH_2_ gel to free drug was 4.6 and 5.7, respectively.

**Figure 9:**
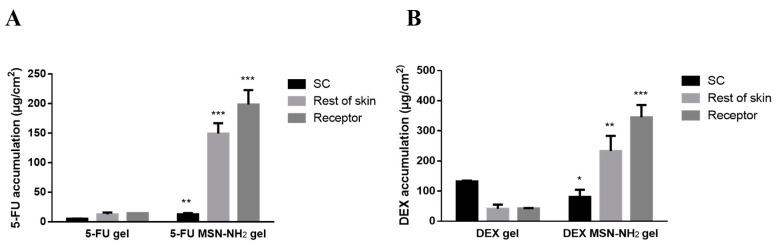
5-FU (**A**) and DEX (**B**) accumulation in different skin layers and receptor media when delivered by MSN-NH2 and free in gel. Data are represented as mean ± SD (*n* = 3). Statistical significance was obtained with *p*-values ≤ 0.05, where * *p* ≤ 0.05, ** *p* ≤ 0.01, *** *p* ≤ 0.001.

For an effective transdermal anticancer activity, chemotherapeutic agents need to exert their action with minimal side effects. This can be achieved if a drug was able to diffuse and accumulate mainly in the epidermis and dermis [54,55]. Therefore, amounts of drugs present in different skin layers were evaluated. The deposition of 5-FU MSN-NH_2_ and DEX MSN-NH_2_ in the remaining skin was significantly higher than the SC layer and when compared to free drugs. Results also showed that there was significant increase in the penetration of 5-FU through the skin when using MSN-NH_2_ in comparison to gel alone (Figure 9A). It is usually less likely for a hydrophilic drug such as 5-FU to remain in the dermis due to the lipophilic nature of skin [54]. However, MSN-NH_2_ seems capable of significantly increasing the accumulation of 5-FU in skin layers to a concentration which was around 10 times higher than control, 149.53 ± 17.4 µg/cm^2^ and 12.71 ± 3.3 µg/cm^2^ for 5-FU MSN-NH_2_ and control, respectively. A significant increase in DEX concentration in the rest of the skin layers was also found with DEX MSN-NH_2_ (233.05 ± 50.3 µg/cm^2^), which was about 5 times higher than control (41.25 ± 14.1 µg/cm^2^) (Figure 9B). MSN-NH_2_ have significantly improved skin permeability of drugs compared to free drug. The improvement in skin permeation could be due to the electrostatic interaction and binding between negatively charged skin and the positively charged amine MSN-NH_2_ surface [56,57]. This interaction could improve the adhesion and contact time of MSN-NH_2_ to the corneocytes (the outermost layer of the epidermis) and provide a larger surface area for a drug to pass through. Once NP accumulate in skin layers, drug release can occur over a prolonged time and diffuse more efficiently throughout the skin and deliver the drug to the affected areas [51,58,59].

### 3.5. In Vitro Cytotoxicity Assay in Melanoma Cells

**Figure 10:**
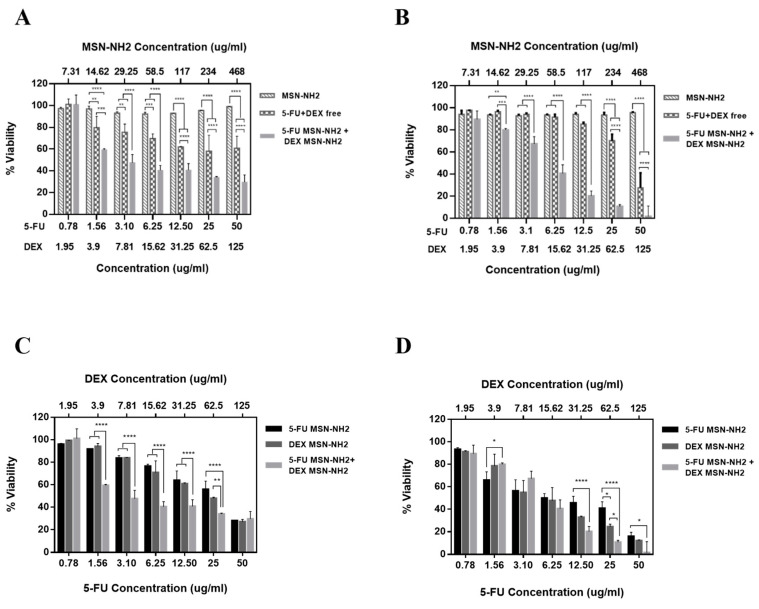
MSN-NH_2_-loaded 5-FU or DEX impairs the viability of HT-144 cells. Cytotoxicity of drug free MSN-NH_2_, 5-FU + DEX free, and 5-FU MSN-NH_2_ + DEX MSN-NH_2_ in HT-144 cells incubated for (**A**) 24 h and (**B**) 48 h. A comparison of the cytotoxicity between 5-FU MSN-NH_2_ alone, DEX MSN-NH_2_ alone, and 5-FU MSN-NH_2_ + DEX MSN-NH_2_ in HT-144 after incubation for (**C**) 24 h and (**D**) 48 h. Data are represented as mean ± SD (*n* = 3). Statistical significance was obtained with *p*-values ≤ 0.05, where * *p* ≤ 0.05, ** *p* ≤ 0.01, *** *p* ≤ 0.001, and **** *p* < 0.0001.

The cytotoxicity of 5-FU/DEX MSN-NH_2_ and 5-FU MSN-NH_2_+DEX MSN-NH_2_ were evaluated in HT-144 melanoma cells for 24, and 48 h. Figure 10A,B shows that drug free MSN-NH_2_ was almost nontoxic to the melanoma cells in the concentration range 0 to 468 µg/mL. Previous reports have shown that silica NPs exert toxicity through the ability of the silanol moiety to generate reactive oxygen species [60]. These species can initiate an intracellular oxidative stress or DNA damage and can bind to proteins and phospholipids on the cellular membrane through the formation of hydrogen bonds or electrostatic interactions [60]. The safety of our developed system can be attributed to the amin surface functionalization, which will mostly shield silanol moieties on the surface on the NPs reducing their chance to interact with the cell, thus inducing less toxicity [61,62].

As for 5-FU, significant difference between 5-FU MSN-NH_2_ and free 5-FU was found only with 50 μg/mL. Maximum cell viability was around 28% and 15% at 24 and 48 h, respectively, for 5-FU MSN-NH_2_ compared to about 40% with free-5-FU at both 24 and 48 h (Figure S2). In contrast, a significant difference in cytotoxicity at 24 h was found between DEX MSN-NH_2_ and free DEX at a lower drug concentration of 31.25 μg/mL. Maximum cell viability at 24 h was 27.05% with DEX MSN-NH_2_ and 35.86% with free DEX using 125 μg/mL. At 48 h, a significant reduction in viability started to appear at DEX concentration of 7.81 μg/mL. Maximum viability of 12.08% with DEX MSN-NH_2_ and 29.74% with free DEX were reported using 125 μg/mL (Figure S3).

To test if treating cells with drug combination using MSN-NH_2_ is advantageous over free drug in terms of cytotoxicity, cells were treated with 5-FU MSN-NH_2_ + DEX MSN-NH_2_ or free 5-FU + DEX. Figure 10A,B indicates that 5-FU MSN-NH_2_ + DEX MSN-NH_2_ started to show a significant reduction in viability compared to free drug combination at 1.56 μg/mL:3.9 μg/mL of 5-FU:DEX. Maximum viability at 24 h using 50 *μ*g/mL:125 *μ*g/mL 5-FU:DEX reached only 29.53% with drugs loaded MSN-NH_2_ which was significantly lower than the combination of free drugs (53.13%) (Figure 10A). At 48 h, the reduction in cell viability was still significant with 5-FU MSN-NH_2_ + DEX MSN-NH_2_ (1.52%) compared to free drug combination (27.54%) at 50 μg/mL:125 μg/mL of 5-F:DEX (Figure 10B). It is noteworthy to mention that there was a significant difference in cell viabilities between mono and combinational treatment using the MSN-NH_2_ at 24 h favoring drugs MSN-NH_2_ combination which was apparent at low drug concentrations (1.56 μg/mL:3.9 μg/mL of 5-FU:DEX) (Figure 10C). However, at high drug concentrations, 50 μg/mL: 125 μg/mL (5-FU:DEX), there was no significant difference in viability. At 48 h, the significant difference between mono and combination therapy occurred only between 5-FU MSN-NH_2_ and 5-FU MSN-NH_2_ + DEX MSN-NH_2_ at 12.5 μg/mL and 25 μg/mL 5-FU. However, at 50 μg/mL, there was no significant difference in viabilities (Figure 10D).

Previous studies showed that 5-FU and DEX treatments are cytotoxic to melanoma cells [38,63]. While each drug alone can induce cytotoxicity to melanoma, it was found that using both free drugs simultaneously can induce cells resistance to treatment mainly owed to the inhibitory effect of DEX on the cytotoxic effect of 5-FU [63]. Figure 10A,B might also indicate that 5 FU can also alter the cytotoxic effect of DEX at least initially at 24 h. These results might partly justify the increase in melanoma cell viability at 48 h after an initial drop at 24 h when cells were treated with free 5-FU + DEX. A similar scenario was not found with cells treated with 5-FU MSN-NH_2_ + DEX MSN-NH_2_. Data suggests that MSN-NH_2_ might have the capability to circumvent DEX induced 5-FU resistance in melanoma cells. The results of this study indicate that treating cells with 5-FU MSN-NH_2_ + DEX MSN-NH_2_ would possibly induce a synergistic cytotoxicity effect against melanoma cells at lower drug concentrations.

It is assumed that the improved cytotoxicity of both drugs is mainly attributed to the improved solubility as well as drug delivery and uptake by the cells compared to the free drug [64]. Having a different loading capacity based on the characteristics of each drugs does not necessarily mean that more drug will give better cytotoxic effect and is all related to the potency of each drug.

## 4. Conclusions

In conclusion, this study demonstrated that surface amino functionalization of MSN was successfully conducted through the utilization of anionic surfactant and APTES and resulted in clear non obstructed mesochannels. These particles were able to efficiently load both hydrophilic and hydrophobic drugs—5-FU and DEX, respectively. Drug release, as well as skin permeability studies under physiological conditions, confirmed the superiority of MSN-NH_2_ to deliver 5-FU and DEX transdermally in a controlled manner. This system showed that it can target skin layers were melanoma usually resides. MSN-NH_2_ has proven to be nontoxic to melanoma cells in vitro but was able to enhance the antiproliferative effect of DEX as well as possibly attenuate DEX induced 5-FU resistance in melanoma. These results therefore indicate that MSN-NH_2_ is a promising delivery vehicle for 5-FU and DEX in the management of melanoma.

## Figures and Tables

**Table 1:** EE%, LC%, and LC in mg mg (Drug)/mg (MSN-NH_2_) of DEX MSN-NH_2_ and 5-FU MSN-NH_2_ using different drug: MSN-NH_2_ ratios.

Ratio Drug:MSN-NH_2_	(Drug:MSN-NH2) (mg in 25 mL)	EE%	LC%	LC mg (Drug)/mg (MSN-NH_2_)
		DEX		
1:5	5:25	47.4 ± 3.15	8.66 ± 0.63	0.087 ± 0.006 mg/1 mg
1:1	25:25	18.89 ± 0.89	15.89 ± 0.88	0.159 ± 0.009 mg/1 mg
5:1	125:25	15.92 ± 0.79	44.72 ± 4.21	0.447 ± 0.042 mg/1 mg
		5-FU		
1:1	25:25	18.01 ± 3.72	15.26 ± 3.13	0.153 ± 0.031 mg/1 mg.
5:1	125:25	3.6 ± 1.4	12.6 ± 5.50	0.126 ± 0.055 mg/1 mg.

**Table 2:** Release kinetics of free formulations, 5-FU MSN-NH_2_, and DEX MSN-NH_2_ loaded using different release kinetic models.

Codes	Zero Order	First Order	Higuchi (Diffusion)	Korsmey-er Peppas	“*n*” Value	K
5-FU gel	0.647	0.604	0.821	0.989	0.159	59.119
5-FU MSN-NH_2_-gel	0.652	0.557	0.833	0.987	0.167	49.671
DEX gel	0.701	0.502	0.964	0.982	0.368	16.602
DEX MSN-NH_2_ gel	0.922	0.701	0.994	0.998	0.602	3.139

**Table 3:** Release kinetics of free formulations, 5-FU MSN-NH_2_, and DEX MSN-NH_2_ loaded using Peppas–Sahlin kinetic model.

Codes	Peppas Sahlin	K1	K2	“*m*” Value
5-FU gel	0.999	61.692	9.170	0.341
5-FU MSN-NH_2_-gel	0.991	42.468	4.783	0.450
DEX gel	0.992	14.249	0.781	0.569
DEX MSN-NH_2_ gel	0.999	3.070	0.452	0.452

**Table 4:** Calculation of permeation parameters.

Formula Code	Flux, J (µg/cm^2^/h)	Permeability Coefficient Kp × 10^−3^ (cm h^−1^)	Lag Time (h)	Enhancement Ratio (ER)
5-FU gel	1.401 ± 0.018	1.75 ± 0.291	0.5	-
5-FU MSN-NH_2_ gel	6.540 ± 0.207	8.22 ± 0.122	0.5	4.668
DEX gel	3.403 ± 0.008	1.70 ± 0.130	0.5	-
DEX MSN-NH_2_ gel	19.330 ± 0.117	38.71 ± 0.297	0.5	5.68

## Supplementary Materials

The following are available online at https://www.mdpi.com/1999-4923/12/11/1035/s1, Table S1. Molar ratio of APTES with respect to other reaction components, Figure S1. Intensity particle size distribution obtained for plain MSN-NH2 (A) DEX MSN-NH2 (B) and 5-FU MSN-NH2 (C), Figure S2. Cytotoxicity of HT-144 cells after the treatment with MSN-NH2, 5-FU free, and 5-FU MSN-NH2 for (A) 24 h, and (B) 48 h, Figure S3. Cytotoxicity of HT-144 cells after the treatment with MSN-NH2, DEX free, and DEX MSN-NH2 for (A) 24 h, and (B) 48 h.
