# Supplementary material for: The Design of Anionic Surfactant-Based Amino-Functionalized Mesoporous Silica Nanoparticles and their Application in Transdermal Drug Delivery
Source: Pharmaceutics. 2020 Oct 29;12(11):1035. doi: 10.3390/pharmaceutics12111035 (PMC7693828; doi:10.3390/pharmaceutics12111035)
Supplement: Supplementary file 1 [file pharmaceutics-12-01035-s001.pdf]

pharmaceutics

Supplementary Materials: The Design of Anionic Surfactant Based Amino-Functionalized Mesoporous Silica Nanoparticles and their Application in Transdermal Drug Delivery

Aliyah A. Almomen , Ahmed M. El-Toni , Mohammed Badran , Adel Alhowyan , Mohd Abul Kalam , Aws Alshamsan and Musaed Alkholief

Table S1. Molar ratio of APTES with respect to other reaction components.

Surfactant	HCl	APTES (vol(μl))		TEOS	Water	Ethanol
0.130	0.013	0.027	(50)	1	217.777	6.705
0.130	0.013	0.041	(75)	1	217.777	6.705
0.130	0.013	0.055	(100)	1	217.777	6.705
0.130	0.013	0.083	(150)	1	217.777	6.705

Physicochemical characteristics of 5-FU/DEX MSN-NH2 gels

All MSN-NH2 gels were investigated by performing their homogeneity using visual inspection to check the prepared gels was homogenous and presence or absence of aggregates. Furthermore, the pH of the formulations was determined using a Mettler Toledo Seven Compact pH meter (Billerica, MA) after dipping directly into the gel product and allowing it to equilibrate for one minute. The viscosity of MSN-NH2 gel in comparison with MSN-NH2 suspension was determined at 25 ºC without dilution using Brookfield viscometer model DV-II with a large sample adaptor connected to Brookfield temperature controller TC-202. (Brookfield Engineering Laboratories, MA, USA) using spindle no. 4S at 4.0 rpm.

Results and discussion

The carbopol 934 gel was selected as hydrogel according to the previously work, which provides good flow properties of NPs for topical and transdermal administration (2). Moreover, 1% w/v of a carbopol gel was prepared and showed proper consistency and a clear appearance (3). MSN-NH2 were added into the carbopol gel and stirred for 24 h to mix uniformly without any aggregation. All formulated gels were described for their cosmetic characters (color, texture and consistency). The gels were light yellowish in color with a smooth homogeneous appearance and texture. The pH value of the formulations ranged from 6.1 to 6.9, which evades the tendency of irritation upon application to the skin (4). The MSN-NH2 suspensions have low viscosity and, therefore, their skin application may be inconvenient. Therefore, carbopol 934 gel was used to increase the viscosity of the NPs for this issue (5). The viscosity of, 5-FU MSN-NH2-gel, DEX MSN-NH2 gel and 5-FU/DEX MSN-NH2 gel were 15520, 17286, and 23151 cP. Therefore, the hydrogels can be applied with MSN-NH2 with good performance and sustained release of the drug. This gel significantly promotes good adhesion, spreading and viability of encapsulated 5-FU and DEX to the skin.

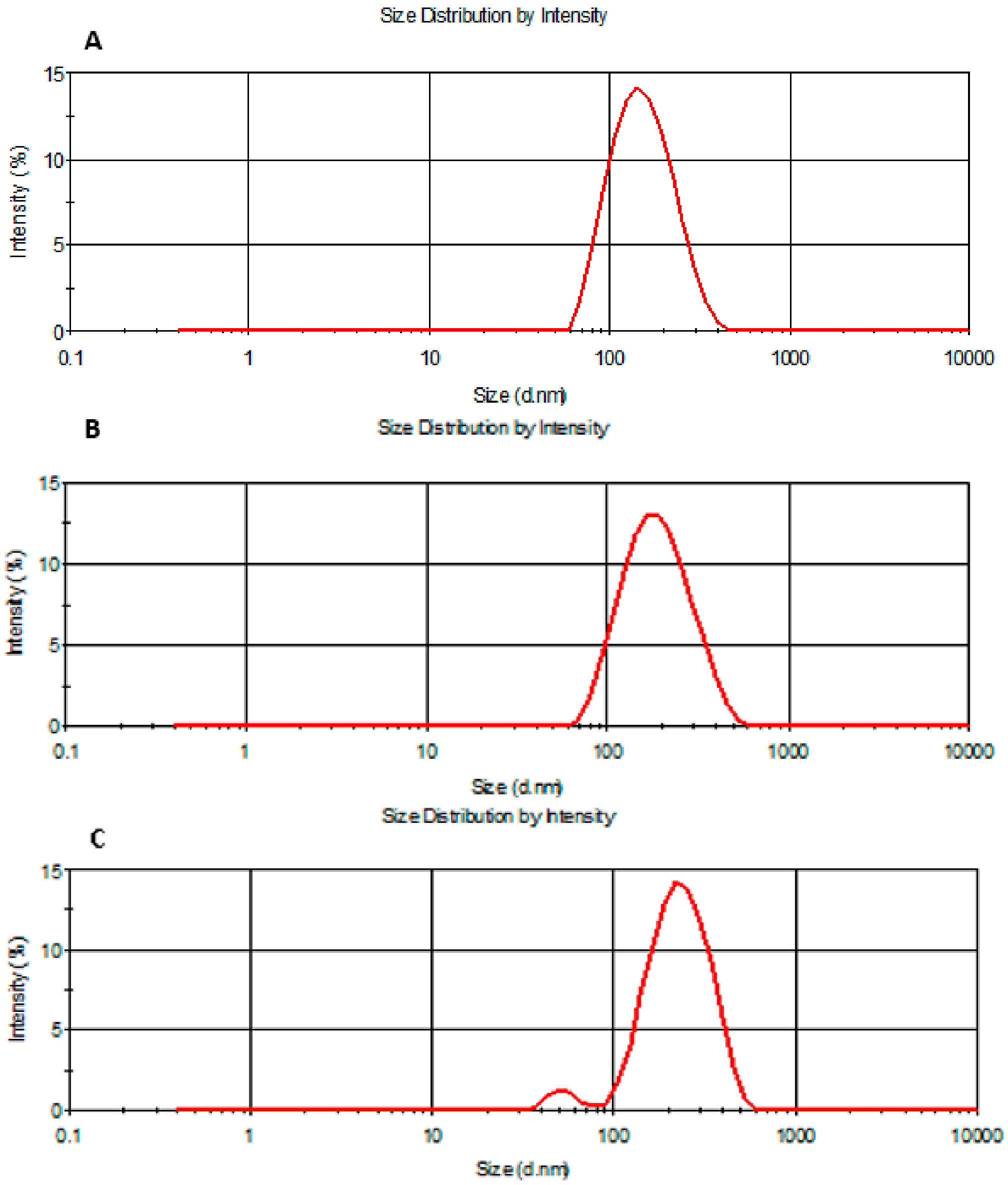

Figure S1. Intensity particle size distribution obtained for plain MSN-NH2 (**A**) DEX MSN-NH2 (**B**) and 5-FU MSN-NH2 (**C**).

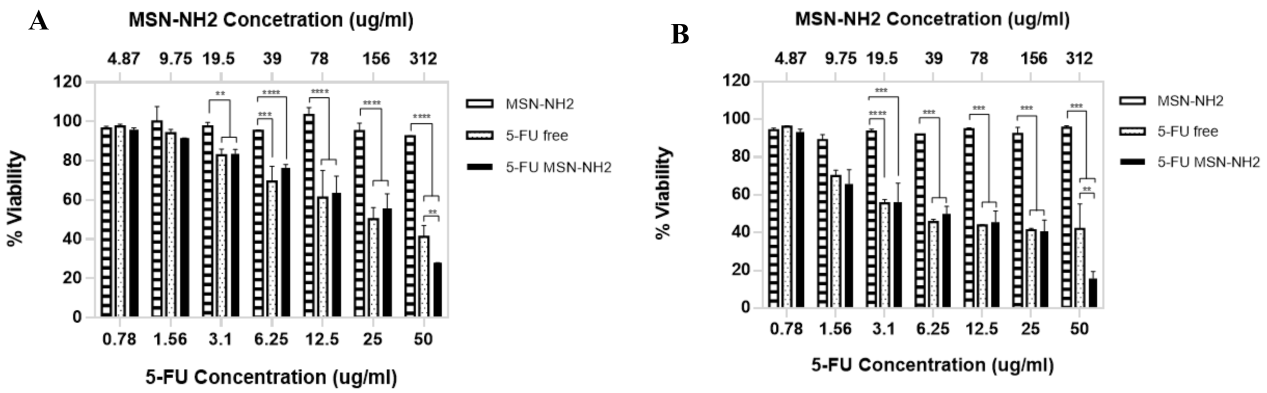

Figure S2. Cytotoxicity of HT-144 cells after the treatment with $MSN-NH_2$, 5-FU free, and 5-FU $MSN-NH_2$ for (**A**) 24 h, and (**B**) 48 h. Data are represented as mean ± SD (*n* = 3).

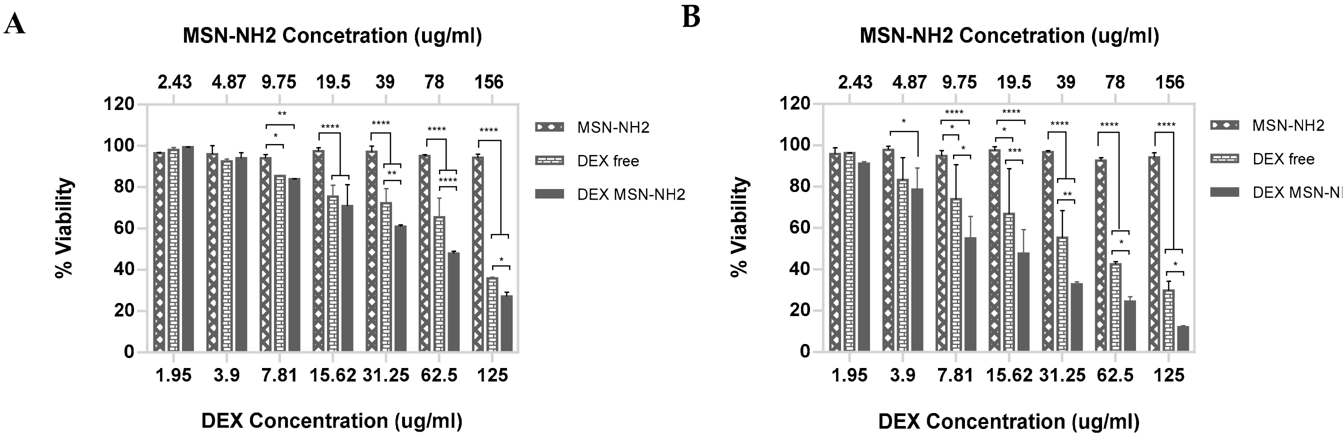

Figure S3. Cytotoxicity of HT-144 cells after the treatment with MSN-NH_2, DEX free, and DEX MSN-NH_2 for (**A**) 24 h, and (**B**) 48 h. Data are represented as mean ± SD (*n* = 3).
